# The α2-HeremansSchmid glycoprotein (AHSG) promotes growth in head and neck squamous cell carcinoma (HNSCC)

**DOI:** 10.1186/1471-2105-14-S17-A7

**Published:** 2013-10-22

**Authors:** Pamela D Thompson, Amos Sakwe, Josiah Ochieng, Siddharth Pratap, Dana R Marshall

**Affiliations:** 1 Department of Biochemistry and Cancer Biology, Meharry Medical College, Nashville, TN, 37208, USA; 2 Bioinformatics and Proteomics Core, Department of Microbiology and Immunology, Meharry Medical College, Nashville, TN, 37208, USA; 3 Department of Pathology, Anatomy and Cell Biology, Meharry Medical College, Nashville, TN, 37208, USA

## Background

AHSG is a calcium-binding glycoprotein synthesized primarily by the liver [1-3] and secreted into serum [1-3]. Primarily known for its role in bone growth and remodeling, it is also reported to be involved in the progression of breast and lung cancer with these cells utilizing the liver synthesized form [1,4,5]. Uniquely, HNSCC cells synthesize their own AHSG, suggesting autocrine signaling fueling cell proliferation and movement. Here we present data showing the presence of AHSG mRNA in HNSCC cell line SQ20B and phenotypic and transcriptomic changes resulting from shRNA knockdown of AHSG protein expression.

## Materials and methods

HNSCC cell line SQ20B was transduced with pGIPZ empty vector or pGIPZ with different AHSG target sequences to generate AHSG deficient cell lines. Depletion of AHSG was confirmed by immunoblot using Meridian AHSG polyclonal antibody and immunocytochemical staining for vector-encoded GFP. To evaluate the physiological loss of AHSG on cell biology, cells were cultured in serum free-media, as serum is a source of AHSG in all animals. Proliferation, migration and invasion were evaluated using standard methodologies. Transcriptomic analysis of cell lines was performed using the Affymetrix Human Gene 2.0 ST chip.

## Results

The role of AHSG in cell proliferation, invasion and migration was evaluated for wildtype SQ20B (SQ20B-WT), SQ20-B with empty vector (SQ20B-EV), SQ20B expressing 50% or 20% of AHSG expressed by SQ20B-EV (SQ20B-AH50 and –AH20 respectively). Decreased AHSG expression resulted in decreased proliferation, migration and invasion of SQ20B cells (Table 1).

**Table 1:** SQ20B-WT, -EV, -AH50 and -AH20 cell lines were evaluated for properties associated with proliferation, migration and invasion. All cell lines were compared to SQ20B-WT

Cell Line	Proliferation	Migration	Invasion
SQ20B-WT	--------------	--------------	-------------
SQ20B-EV	--------------	--------------	-------------
SQ20B-AH50	--------------	p < 0.05	p < 0.05
SQ20B-AH20	p < 0.05	p < 0.009	p < 0.009

Transcriptomic analysis identified genes associated with cancer and cellular movement as two of the top biofunction categories associated with AHSG molecular function.

**Table 2:** Top biofunctions calculated (Ingenuity Pathways Analysis) from gene list generated by comparison of SQ20B-AH20 and SQ20B-WT for genes differentially expressed at p< 0.05 (Benjamini and Hochberg multiple testing correction) and fold difference ≥1.5

Diseases and Disorders	p-value	# Molecules
Dermatological Diseases and Conditions	2.00E-08 – 1.47E-02	59
Cancer	1.90E -07 – 1.55E-02	103
Reproductive System Disease	2.31E -07 – 1.48E-02	54
Endocrine System Disorders	3.17E-07 – 2.29E-03	28
Inflammatory Responses	7.32E-06 – 1.62E-02	48

**Molecular and Cellular Functions**		

Cellular Movement	5.75E-06 – 1.62E-02	61
Lipid Metabolism	6.04E-05 – 1.47E-02	24
Molecular Transport	6.04E-05 – 1.59E-02	41
Small Molecule Biochemistry	6.04E-05 – 1.51E-02	36
Cell-To-Cell Signaling and Interaction	8.26E-05 – 1.59E-02	46

## Conclusions

AHSG affects *in vitro* cellular properties associated with metastatic potential *in vivo.* Transcriptomic analysis has identified highly relevant transcriptomic programs of expression that will highlight new cellular processes that are likely associated with metastasis of HNSCC.
